# Intra‐ and inter‐session reliability and repeatability of ^1^H magnetic resonance spectroscopy for determining total creatine concentrations in multiple brain regions

**DOI:** 10.1113/EP092252

**Published:** 2024-12-20

**Authors:** Jedd Pratt, James McStravick, Aneurin J. Kennerley, Craig Sale

**Affiliations:** ^1^ Department of Sport and Exercise Sciences Manchester Metropolitan University Institute of Sport Manchester UK; ^2^ Department of Allied Health Professions and Sport and Exercise Science, School of Human and Health Sciences University of Huddersfield Huddersfield UK

**Keywords:** brain, creatine, magnetic resonance spectroscopy, reliability, reproducibility

## Abstract

Using proton magnetic resonance spectroscopy (^1^H MRS) to determine total creatine (tCr) concentrations will become increasingly prevalent, as the role of creatine (Cr) in supporting brain health gains interest. Methodological limitations and margins of error in repeated ^1^H MRS, which often surpass reported effects of supplementation, permeate existing literature. We examined the intra‐ and inter‐session reliability and repeatability of ^1^H MRS for determining tCr concentrations across multiple brain regions (midbrain, visual cortex and frontal cortex). Eighteen healthy adults aged 20–32 years were recruited (50% female; *n* = 14 intra‐session; *n* = 15 inter‐session). ^1^H Magnetic resonance imaging and spectroscopy were completed at 3 T. Intra‐session analyses involved repeated ^1^H MRS of the midbrain, visual cortex and frontal cortex without participant or voxel repositioning, whereas inter‐session analyses involved measurements of the same regions, but with participant and voxel repositioning between repeated measurements. The ^1^H MRS data (174 spectra) were analysed using TARQUIN and OSPREY, and voxel fractions (grey/white matter and CSF) were determined using segmentation. Our findings show that tCr concentrations can be determined reliably and repeatably using ^1^H MRS, within an error of <2%, and that large inter‐regional differences in tCr concentration are present in the human brain. We provide new minimum detectable change data for tCr concentrations, a detailed discussion of the inherent error sources in repeated ^1^H MRS, including the substantial effect of the analysis package on tCr quantification, and suggestions for how these should be managed to improve the interpretability and clinical value of future research. More studies are needed to determine whether our findings can be replicated in other centres and different populations.

## Abstract (highlights)

- **What is the central question of this study?**
  What are the intra‐ and inter‐session reliability and repeatability of ^1^H magnetic resonance spectroscopy for determining total creatine concentrations in the human brain, and does the choice of analysis package matter?
- **What is the main finding and its importance?**
^1^H Magnetic resonance spectroscopy can determine total creatine concentrations accurately and repeatably across multiple brain regions, within an error of <2%, although the inherent error sources in repeated ^1^H magnetic resonance spectroscopy need to be managed carefully. We discuss these error sources, including the choice of analysis package, and provide suggestions for how these should be managed to improve the interpretability and quality of future work.

## 1 INTRODUCTION

Proton magnetic resonance spectroscopy (^1^H MRS) has emerged as a promising non‐invasive method of determining metabolite concentrations in the human brain (Wilson et al., 2019). The clinical value of ^1^H MRS depends upon the accuracy and precision of repeated metabolite measurements and its demonstrable ability to quantify responses to an intervention. Without this information, changes in metabolite concentrations that might be associated with disease, challenges to cognitive function or responses to supplementation, for example, cannot be distinguished confidently from changes arising from systemic variability in ^1^H MRS. Although many studies have provided ^1^H MRS reliability and repeatability data for certain metabolites, such as GABA (Baeshen et al., 2020; Brix et al., 2017; Elsaid et al., 2023; Near et al., 2013), fewer have reported such data for creatine (Cr) (Terpstra et al., 2016; Wijtenburg et al., 2014), which, in ^1^H MRS, is representative of the total Cr pool [tCr; i.e., the sum of free Cr and phosphorylcreatine (PCr)].

Creatine serves an indispensable role in brain energetics by supporting the resynthesis and maintenance of ATP concentrations, which might become increasingly important during metabolically demanding periods (e.g., ageing, neurodegenerative diseases, mild traumatic brain injury, sleep deprivation) (Candow et al., 2023; Forbes et al., 2022). Consequently, increasing attention has been given to determining whether brain Cr concentrations differ according to these heightened states of metabolic demand and whether oral ingestion of commercially produced Cr can increase brain Cr levels and subsequently improve cognitive health. Although some studies have reported differences in brain Cr concentrations between several physiological states, including ‘typical’ ageing, schizophrenia and Alzheimer's disease (ranging between 14.2% and 21%) (Lind et al., 2020; Ongür et al., 2009; Pfefferbaum et al., 1999), and increases in brain Cr following supplementation (ranging between 3.4% and 9.2%) (Dechent et al., 1999; Gordji‐Nejad et al., 2024; Lyoo et al., 2003; Pan & Takahashi, 2007; Turner et al., 2015), others have reported no differences between phenotypes or changes following supplementation (Bender et al., 2005; Merege‐Filho et al., 2017; Solis et al., 2017; Wilkinson et al., 2006; Yazigi Solis et al., 2014). Besides the overall scarcity and incongruity of existing literature, a somewhat larger issue is the apparent lack of consideration of existing studies for the accuracy, or potential inaccuracy, of ^1^H MRS in detecting changes in Cr, which is ultimately needed to underpin the integrity of findings.

There are several potential sources of error in repeated ^1^H MRS acquisition, which can be broadly categorized as follows: (1) repeated voxel localization; (2) participant compliance; (3) physiological variation; and (4) data acquisition and analysis procedures. Typically, ^1^H MRS requires placing a voxel of interest (VOI) in a selected brain region, with the precise three‐dimensional placement being operator dependent. Positioning the VOI in the same location for repeated measurements is challenging and, when considered in parallel with data indicating that Cr concentrations can differ according to brain region (Minati et al., 2010; Rodríguez‐Nieto et al., 2023), is a likely source of error between repeated measurements. The accuracy of voxel placement is also reliant upon the ability of participants to remain still during the procedure, because movement can lead to inconsistent measurement locations, which can affect voxel composition [i.e., the fraction of CSF, grey matter (GM) and white matter (WM)] and, potentially, metabolite concentration.

Once acquired, ^1^H MRS data can be challenging to analyse; consequently, metabolite concentrations are often expressed as a ratio, such as tCr/choline or tCr/*N*‐acetylaspartate, rather than an absolute value (Ferguson et al., 2002; Gordji‐Nejad et al., 2024; Lyoo et al., 2003), which precludes an accurate detection of change in tCr concentration. Importantly, technological developments, including the establishment of automated analysis tools [such as totally automatic robust quantitation in NMR (TARQUIN) (Wilson et al., 2011) and open‐source processing, reconstruction and estimation of magnetic resonance spectroscopy data (OSPREY) (Oeltzschner et al., 2020)], have allowed absolute metabolite quantification to be more accessible, and their use might enhance the overall reliability and repeatability of ^1^H MRS data. It is also noteworthy, however, that although inter‐software comparisons are somewhat scarce, particularly for tCr determination, emerging data suggest that even when identical MRS data are analysed, large differences in metabolite concentration can be observed according to the analysis software used (Zöllner et al., 2021). As such, there remains an urgent need to improve our understanding of the effect of the analysis package on absolute tCr concentration determination and the reliability and repeatability of these data.

The importance of addressing the inherent sources of error in ^1^H MRS is demonstrated by substantial margins of error between repeated metabolite measurements in existing reliability and repeatability studies. Early studies using 1.5 T suggested that changes in tCr concentration needed to be close to 20% (Brooks et al., 1999; Charles et al., 1996; Li et al., 2003; Marshall et al., 1996) to be detected by ^1^H MRS, although, encouragingly, more recent studies using higher magnetic fields (3 or 7 T) have reported smaller error bands, with mean coefficients of variation (CV) and absolute differences between scans ranging between ∼5% and ∼10% (Okada et al., 2021; Stephenson et al., 2011; Terpstra et al., 2016; Wang et al., 2024; Wijtenburg et al., 2013). The quality of these data is, however, impeded by small sample sizes (*n* = 3–6) (Terpstra et al., 2016; Wang et al., 2024; Wijtenburg et al., 2013), lack of consideration of multiple brain regions (Okada et al., 2021; Stephenson et al., 2011; Wang et al., 2024) and, importantly, methodological design that precludes the ability to decipher the error specifically related to repeated ^1^H MRS measurement, rather than biological variation in tCr that might occur during follow‐up periods (i.e., repeated scans are not immediate, but separated by ≤10 days; Stephenson et al., 2011; Terpstra et al., 2016; Wang et al., 2024; Wijtenburg et al., 2013). It is particularly noteworthy that although the accuracy of ^1^H MRS has improved in recent years, the majority of existing data suggest margins of measurement error that broadly encompass the changes in brain tCr concentrations reported in supplementation trials to date (ranging between 3.4% and 9.2%) (Dechent et al., 1999; Gordji‐Nejad et al., 2024; Lyoo et al., 2003; Pan & Takahashi, 2007; Turner et al., 2015). Therefore, for accurate differentiation of an intervention (e.g., supplementation) effect from inherent measurement variability, further consideration needs to be given to the reliability and repeatability of ^1^H MRS.

Herein, our aim was to determine the intra‐ and inter‐session reliability and repeatability of ^1^H MRS for determining tCr concentrations in multiple brain regions of healthy young adults, using two state‐of‐the‐art analysis packages (i.e., TARQUIN and OSPREY). Three brain regions were selected [midbrain (MB), visual cortex (VC) and frontal cortex (FC)] owing to their relevance to neurological diseases and movement disorders (Caligiore et al., 2016; Takakusaki, 2013; Wu et al., 2023).

## 2 MATERIALS AND METHODS

### 2.1 Ethical approval

Ethical approval was granted by Manchester Metropolitan University's research ethics committee (approval number 47922), and written informed consent was obtained from each participant prior to enrolment. The study conformed to the standards set by the *Declaration of Helsinki*.

### 2.2 Study sample and design

**FIGURE 1:**
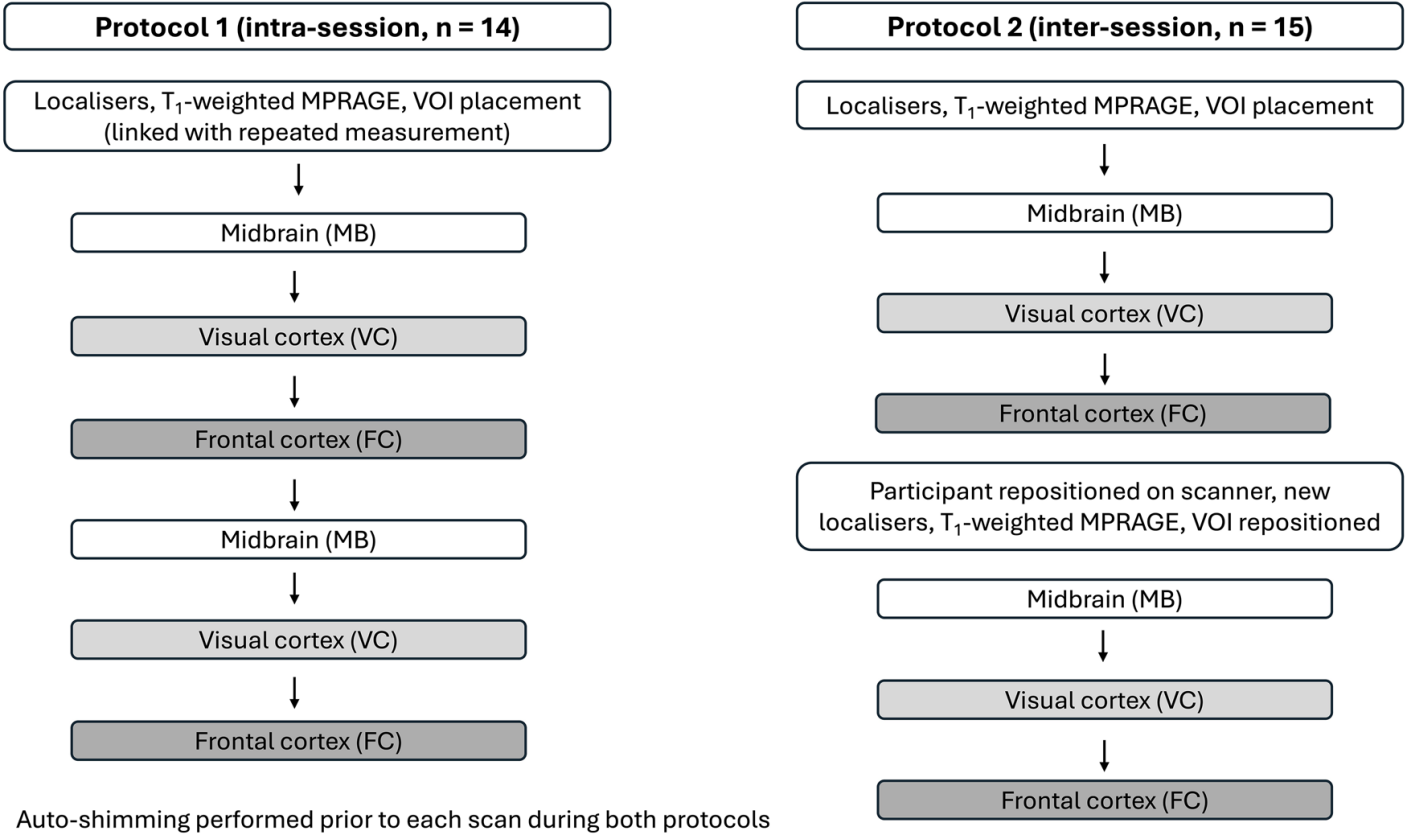
Overview of intra‐session and inter‐session protocols. MPRAGE, magnetization prepared rapid gradient echo; VOI, voxel of interest.

Eighteen healthy young adults aged between 20 and 32 years took part in this study (mean age = 25.8 ± 3.0 years; 50% female). Two protocols were integrated into the study design to allow for the assessment of intra‐ and inter‐session reliability and repeatability of ^1^H MRS. Protocol 1 (intra‐session) lasted ∼45 min and involved back‐to‐back measurements of the MB, VC and FC, without removing the participant from the scanner or repositioning the VOI (VOI location was copied from the first to the second measurement) (Figure 1). Protocol 2 (inter‐session) lasted ∼60 min and also involved two measurements of the MB, VC and FC, but incorporated a brief break between repeated measurements, during which the participant was removed fully from the scanner and repositioned back in the scanner. The measurement taken after the break was treated as a new visit and, as such, involved the acquisition of a new structural scan and the repositioning of the VOI for each brain region as close to the location of the first measurement as possible (Figure 1). Anatomical landmarks and the previous measurement image were used to enhance the accuracy of repeated VOI placement. All sessions were conducted by an experienced MR operator. Importantly, the inter‐session aspect of this study was designed to allow for the specific variance associated with repeated ^1^H MRS to be distinguished. Separating repeated measurements with a short break, rather than long break as seen in previous studies (Terpstra et al., 2016; Wang et al., 2024; Wijtenburg et al., 2013), allowed the complexities associated with subject and voxel repositioning to be captured, while avoiding potential inaccuracies in repeated ^1^H MRS variability estimates that could be caused by biological variation in Cr that might occur over longer periods. A total of 14 participants completed the intra‐session protocol, and 15 participants completed the inter‐session protocol (11 of these participants completed both protocols).

### 2.3 Magnetic resonance acquisition

**FIGURE 2:**
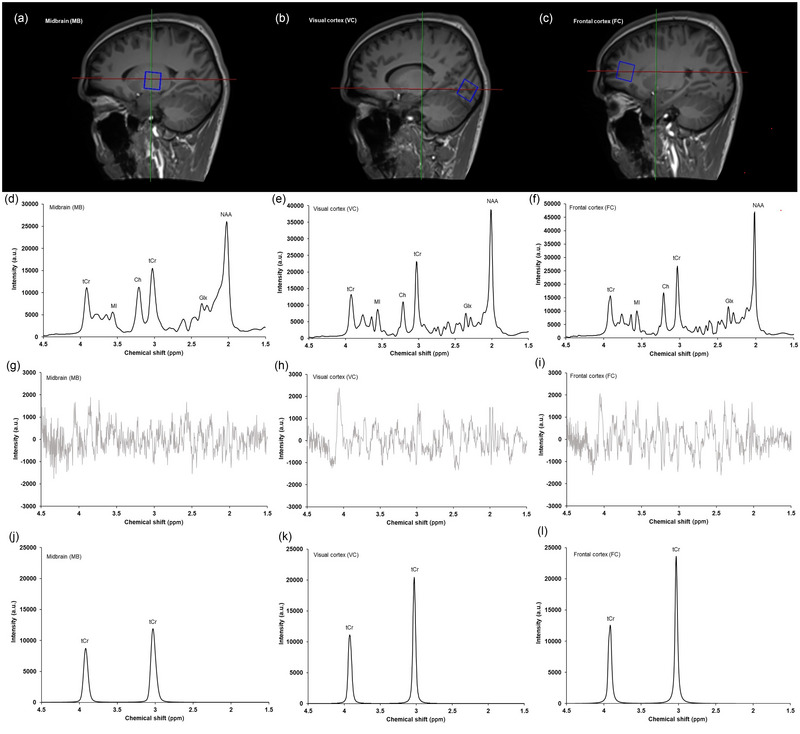
Representative voxel positioning (a–c), fitted spectra (d–f), residuals (g–i) and model fits for total creatine (j–l), acquired from the midbrain (a, d, g, j), visual cortex (b, e, h, k) and frontal cortex (c, f, i, l). Metabolites: Ch, choline; Glx, glutamate–glutamine complex; MI, *myo*‐inositol; NAA, *N*‐acetylaspartate; tCr, total creatine.

All MR imaging and spectroscopy were performed on a 3 T Siemens MAGNETOM Vida (Siemens Healthineers, Erlangen, Germany), using a 20‐channel receive‐only head coil. Each participant was positioned carefully on the scanner to ensure their comfort and improve compliance with the protocol. Additionally, cushioning was positioned around the participant's head to dampen head movements during the scan duration. Prior to ^1^H MRS, a comprehensive localization protocol was used to facilitate accurate voxel placement. This included two localizers integrating distortion correction and a high‐resolution T_1_‐weighted magnetization prepared rapid gradient echo (MPRAGE) sequence acquired in the sagittal plane [(repetition time) TR = 2100 ms; (echo time) TE = 2.58 ms; flip angle = 8°; slice thickness 1 mm; slice number = 176; (field of view) FoV = 250 mm; voxel size = 1 mm × 1 mm × 1 mm]. Sagittal images were converted into axial and coronal planes, and all three planes were used as references to position the VOI in the MB, VC and FC (Figure 2), according to anatomical landmarks and the previous measurement (for the inter‐session protocol). The VOI was positioned in the left hemisphere of every participant. The ^1^H MRS was acquired from a 20 mm × 20 mm × 20  mm voxel using a point resolved spectroscopy (PRESS) sequence [(repetition time) TR = 2000 ms; (echo time) TE = 30 ms; 130 averages; bandwidth = 1200 Hz]. Siemens brain automated static magnetic field (B_0_) shimming and standard chemical shift selective (CHESS) suppression with 50 Hz bandwidth were used. Immediately after the water‐suppressed acquisition, using the same parameters, 10 water‐unsuppressed averages were taken, which were later used for eddy‐current correction and absolute tCr quantification (detailed in Section 2.4). The total acquisition time per spectrum was 5 min.

### 2.4 Data processing, quality of spectra, CSF contamination and Cr quantification

The ^1^H MRS data were processed and analysed with TARQUIN (v.4.3.10) and OSPREY (v.2.6.0), using the water‐unsuppressed signal as a reference to determine absolute tCr concentrations.

TARQUIN is an established automated analysis tool, comparable to LCModel (Provencher, 1993), that uses time‐domain truncation to mitigate baseline interference and internal basis sets to determine metabolite concentrations (Wilson et al., 2011). Following imputation of water‐unsuppressed and water‐suppressed ^1^H MRS data into TARQUIN, an eddy‐current correction and lipid filtering were applied prior to model fitting to enhance the quality of data, as recommended by a recent consensus statement on ^1^H MRS methodology in the brain (Wilson et al., 2019).

OSPREY is a state‐of‐the‐art MATLAB‐based toolbox that facilitates processing and analysis of metabolite concentrations in vivo. As with TARQUIN, water‐unsuppressed and water‐suppressed data were imputed, and an eddy‐current correction and lipid filter were applied prior to tCr quantification.

Zero‐ and first‐order phase corrections and baseline corrections were performed in TARQUIN and OSPREY. Default standard ^1^H MRS basis sets were used for metabolite quantification in both software packages, which were based on Siemens PRESS and included the following metabolites in the quantification process: alanine, aspartate, creatine, GABA, glucose, glutamine, glutamate, glycerophosphochlorine, guanidinoacetate, *myo*‐inositol, lactate, *N*‐acetylaspartate, *N*‐acetylaspartylglutamate, phosphorylcholine, phosphorylcreatine, scyllo‐inositol and taurine. Given that Cr and PCr both resonate at 3.03 and 3.92 ppm, it is not possible to distinguish Cr from PCr confidently using ^1^H MRS at 3 T. Therefore, Cr and PCr were fitted together, and the sum of the two peaks was considered as the tCr value. Following the consensus guidelines for reporting standards in MRS (Lin et al., 2021), spectra quality metrics, including water peak full width at half‐maximum (FWHM), signal‐to‐noise ratio (SNR), Cramér–Rao lower bound (CRLB) and overall standard deviation (SD) of residuals, were determined during the model‐fitting procedure for each spectrum. The CSF, grey matter (BM) and white matter (WM) fractions of each voxel for every participant were calculated using SPM12 segmentation within the OSPREY interface (Oeltzschner et al., 2020). The segmentation process was completed separately from the quantification process and, as such, had no effect on metabolite quantification in either TARQUIN or OSPREY. Inter‐session, intra‐subject displacement of the VOI centre was determined for each brain region for every participant using MRIcro.

### 2.5 Statistical analyses

Intra‐ and inter‐session reliability and repeatability were evaluated using Pearson's correlation coefficient (*r*), coefficient of variation (CV), intraclass correlation coefficient (ICC) and Bland–Altman (BA) plots. Thresholds for interpreting *r* were as follows: 0–0.09, no correlation; 0.1–0.39, weak correlation; 0.40–0.69, moderate correlation; 0.7–0.89, strong correlation; and ≥0.9, very strong correlation. Thresholds for interpreting ICC were as follows: <0.5, poor reliability; 0.5–0.74, moderate reliability; 0.75–0.89, good reliability; and ≥0.9, excellent reliability. Thresholds for interpreting CV were as follows: <10%, low; 10%–19%, moderate; 20%–29%, high; and ≥30%, very high. A low CV indicates lower measurement variability relative to the mean, hence good repeatability, whereas a high CV indicates greater measurement variability, hence poor repeatability. Minimum detectable changes (MDCs) were calculated for each brain region. Student's paired *t*‐tests were performed to examine intra‐individual, intra‐ and inter‐session differences in absolute tCr concentration. ANOVA was used to determine differences in spectral quality metrics and tCr concentrations according to brain region and analysis package, using Tukey's honest significance difference (HSD) tests to determine specific areas of significance. The threshold for statistical significance was set at *P *< 0.05. Statistical analyses were performed using SPSS (v.28; IBM SPSS Inc., Chicago, IL, USA), and data visualizations were developed using GraphPad Prism (v.9.3.1; GraphPad, San Diego, CA, USA).

## 3 RESULTS

### 3.1 Quality of spectra, voxel fractions and relocalization accuracy, and correlation with tCr concentration

**FIGURE 3:**
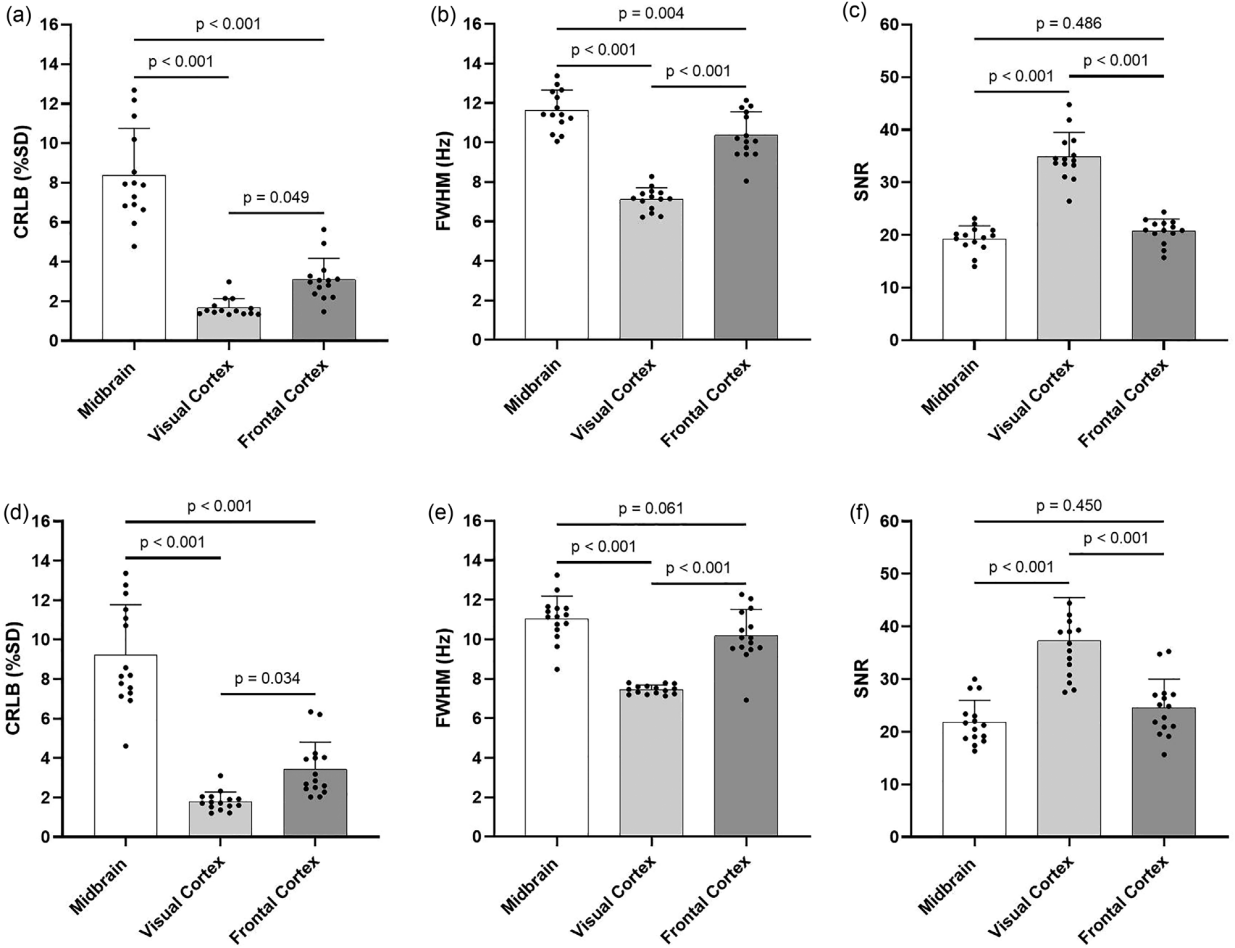
Inter‐regional differences in the average quality metrics of the spectra acquired during the intra‐session (a–c) and inter‐session protocols (d–f). Abbreviations: CRLB, Cramér–Rao lower bound; FWHM, full width at half‐maximum; SNR, signal‐to‐noise ratio.

A total of 174 spectra were acquired, consisting of 84 for intra‐session analyses (28 per brain region) and 90 for inter‐session analyses (30 per brain region). Representative spectra for the MB, VC and FC are displayed in Figure 2. Example raw spectra for each brain region are presented in Figure S1. Average spectral quality indicators according to brain region and protocol design are presented in Figure 3. In the intra‐session analyses, the CRLB and water FWHM in the VC were significantly lower than in the FC (*P* = 0.049 and *P* < 0.001) and MB (both *P* < 0.001), and the SNRs in the VC were significantly higher than those in the FC and MB (both *P* < 0.001). Likewise, in the inter‐session analyses, the CRLB and water FWHM in the VC were significantly lower than in the FC (*P* = 0.034 and *P* < 0.001) and MB (both *P* < 0.001), and the SNRs in the VC were significantly higher than in the FC and MB (both *P* < 0.001) (Figure 3). Importantly, although inter‐regional differences were shown, the average quality metrics for all brain regions were of suitably high quality for analyses (Öz et al., 2020). A full list of the quality metrics for all acquired spectra can be found in the Supplementary Material. Overall, the SDs of fit residuals were 672.9 ± 15.6, 448.5 ± 10.4 and 592.8 ± 14.7 a.u., for the MB, VC and FC, respectively.

**FIGURE 4:**
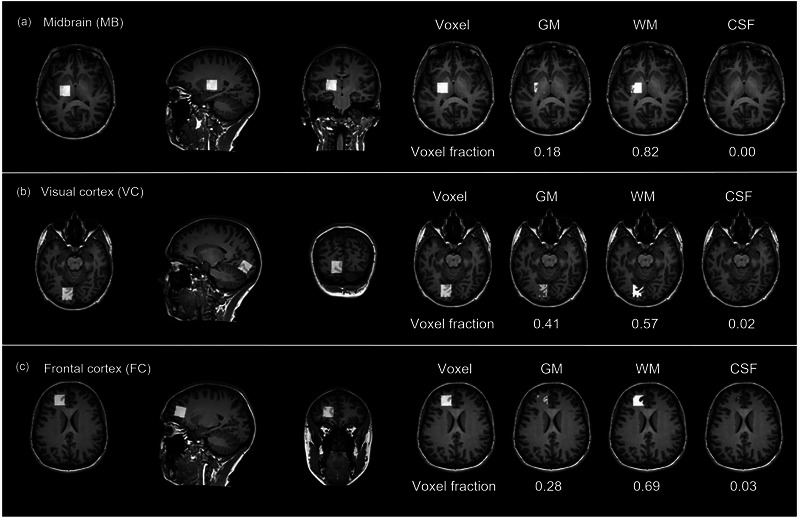
Example voxel fractions of grey matter (GM), white matter (WM) and CSF in the midbrain (a), visual cortex (b) and frontal cortex (FC).

Across the full sample, mean voxel fractions of GM, WM and CSF were 14.7% ± 0.6%, 84.7% ± 0.7% and 0.6% ± 0.4% in the MB, 45.5% ± 1.4%, 50.8% ± 2.0% and 3.7% ± 0.8% in the VC and 29.2% ± 2.9%, 64.8% ± 5.4% and 6.0% ± 0.7% in the FC, respectively (example voxel fractions are presented in Figure 4). Mean differences in GM, WM and CSF between repeated inter‐session measurements were 2.6% ± 0.4%, 2.5% ± 0.5% and 0.2% ± 0.1% in the MB, 3.5% ± 0.6%, 4.1% ± 0.8% and 0.9% ± 0.3% in the VC and 3.9% ± 0.7%, 4.2% ± 0.8% and 1.3% ± 0.4% in the FC, respectively. Change in tissue and CSF fractions between repeated measurements was not correlated with inter‐session differences in tCr concentrations in any brain region (Table S1), hence no corrections were performed.

Mean differences in CRLB, water FWHM and SNR between repeated intra‐session measurements were 30.0% ± 6.5%, 3.2% ± 0.8% and 13.6% ± 2.5% in the MB, 14.3% ± 4.6%, 1.3% ± 0.3% and 9.9% ± 3.1% in the VC and 21.6% ± 5.9%, 5.1% ± 1.2% and 20.6% ± 4.6% in the FC, respectively. Mean differences in CRLB, water FWHM and SNR between repeated inter‐session measurements were 30.0% ± 5.3%, 8.6% ± 1.4% and 23.8% ± 5.8% in the MB, 17.8% ± 3.7%, 3.9% ± 1.1% and 14.6% ± 4.3% in the VC and 36.4% ± 12.4%, 7.5% ± 1.5% and 28.5% in the FC, respectively. No significant correlations were shown between intra‐ or inter‐session changes in CRLB, water FWHM or SNR and intra‐ or inter‐session differences in tCr concentrations (Tables S2 and S3). The mean inter‐session, intra‐participant displacement of the VOI centre was 2.1 ± 0.21 mm. The degree of displacement was 1.76 ± 0.37 mm in the MB, 2.00 ± 0.30 mm in the VC and 2.71 ± 0.40 mm in the FC. No significant correlations were shown between the degree of VOI displacement and inter‐session differences in tCr (Table S4).

### 3.2 Intra‐session reliability and repeatability of ^1^H MRS

**FIGURE 5:**
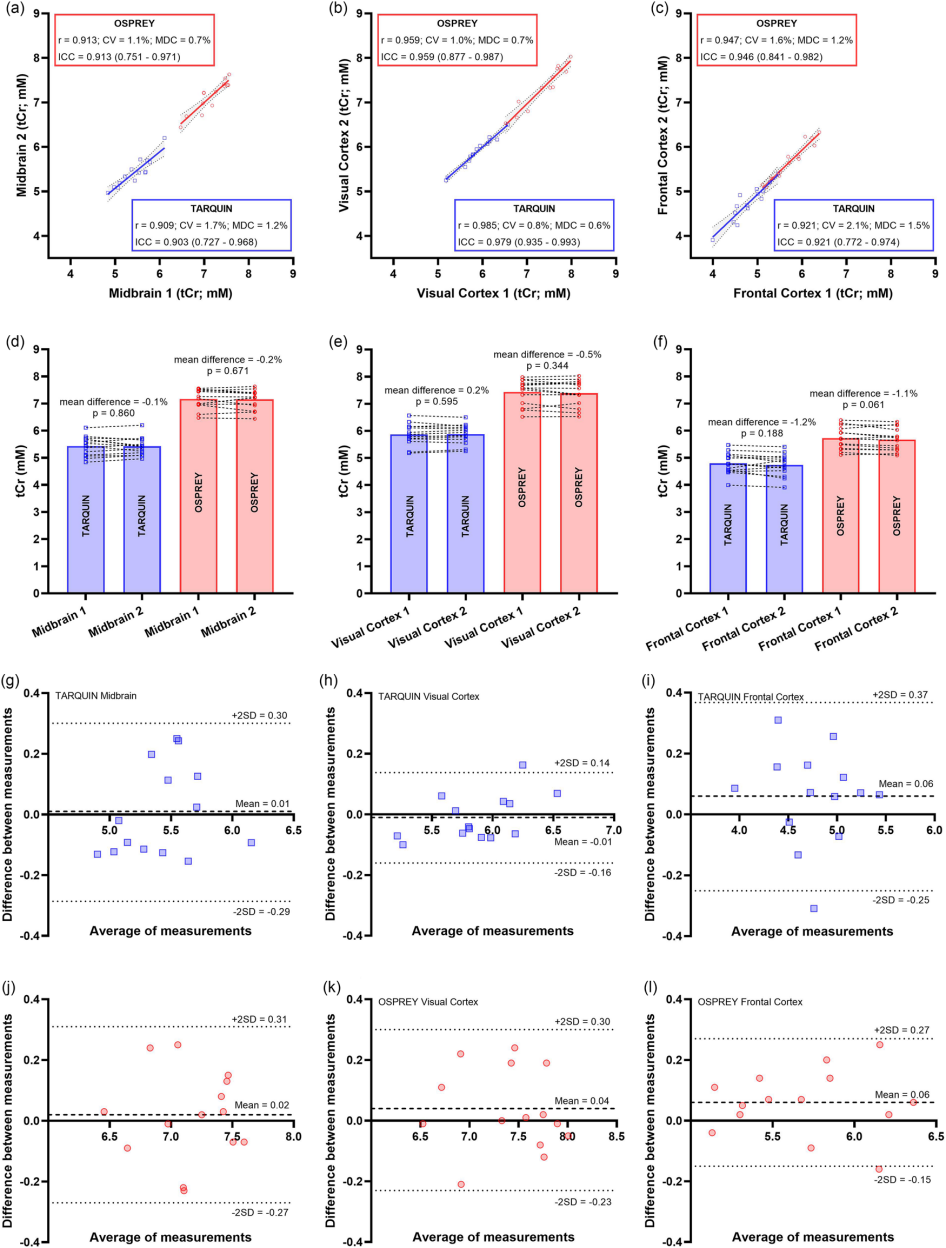
Intra‐session reliability and repeatability of ^1^H magnetic resonance spectroscopy for repeated total creatine (tCr) quantification in the midbrain (a, d, g, j), visual cortex (b, e, h, k) and frontal cortex (c, f, i, l), using TARQUIN (blue) or OSPREY (red). Pearson's correlation coefficients (*r*), coefficients of variation (CV), intra‐class correlation coefficients (ICC) and minimum detectable change (MDC) are shown (a–c), with the mean absolute differences (d–f) and Bland–Altman plots (g–l).

Very strong Pearson's correlation coefficients were shown between repeated intra‐session tCr measurements for all brain regions, using either TARQUIN or OSPREY (for TARQUIN: MB, *r* = 0.909; VC, *r* = 0.985; and FC, *r* = 0.921; and for OSPREY: MB, *r* = 0.913; VC, *r* = 0.959; and FC, *r* = 0.947; all *P* < 0.001; Figure 5). No significant differences in mean absolute tCr concentrations were shown between repeated intra‐session measurements in any brain region, regardless of the analysis package (for TARQUIN: MB measurement 1 vs. MB measurement 2 = −0.1%, *P* = 0.860; VC measurement 1 vs. VC measurement 2 = 0.2%, *P* = 0.595; and FC measurement 1 vs. FC measurement 2 = −1.2%, *P* = 0.168; and for OSPREY: MB measurement 1 vs. MB measurement 2 = −0.2%, *P* = 0.671; VC measurement 1 vs. VC measurement 2 = −0.5%, *P* = 0.344; and FC measurement 1 vs. FC measurement 2 = −1.1%, *P* = 0.061). For TARQUIN, the corresponding CVs were 1.7%, 0.8% and 2.1%, and the ICCs were 0.903 [95% confidence interval (CI) = 0.727–0.968, *P* < 0.001], 0.979 (95% CI = 0.935–0.993, *P* < 0.001) and 0.921 (95% CI = 0.772–0.974, *P* < 0.001) for the MB, VC and FC, respectively. For OSPREY, the corresponding CVs were 1.1%, 1.0% and 1.6%, and the ICCs were 0.913 (95% CI = 0.751–0.971, *P* < 0.001), 0.959 (95% CI = 0.877–0.987, *P* < 0.001) and 0.946 (95% CI = 0.841–0.982, *P* < 0.001) for the MB, VC and FC, respectively. No systematic trends in measurement bias were shown on the Bland–Altman plots, further indicating strong agreement between repeated tCr measurements (Figure 5). Minimum detectable changes were 1.2%, 0.6% and 1.5% (TARQUIN) and 0.7%, 0.7% and 1.2% (OSPREY) for the MB, VC and FC, respectively.

### 3.3 Inter‐session reliability and repeatability of ^1^H MRS

**FIGURE 6:**
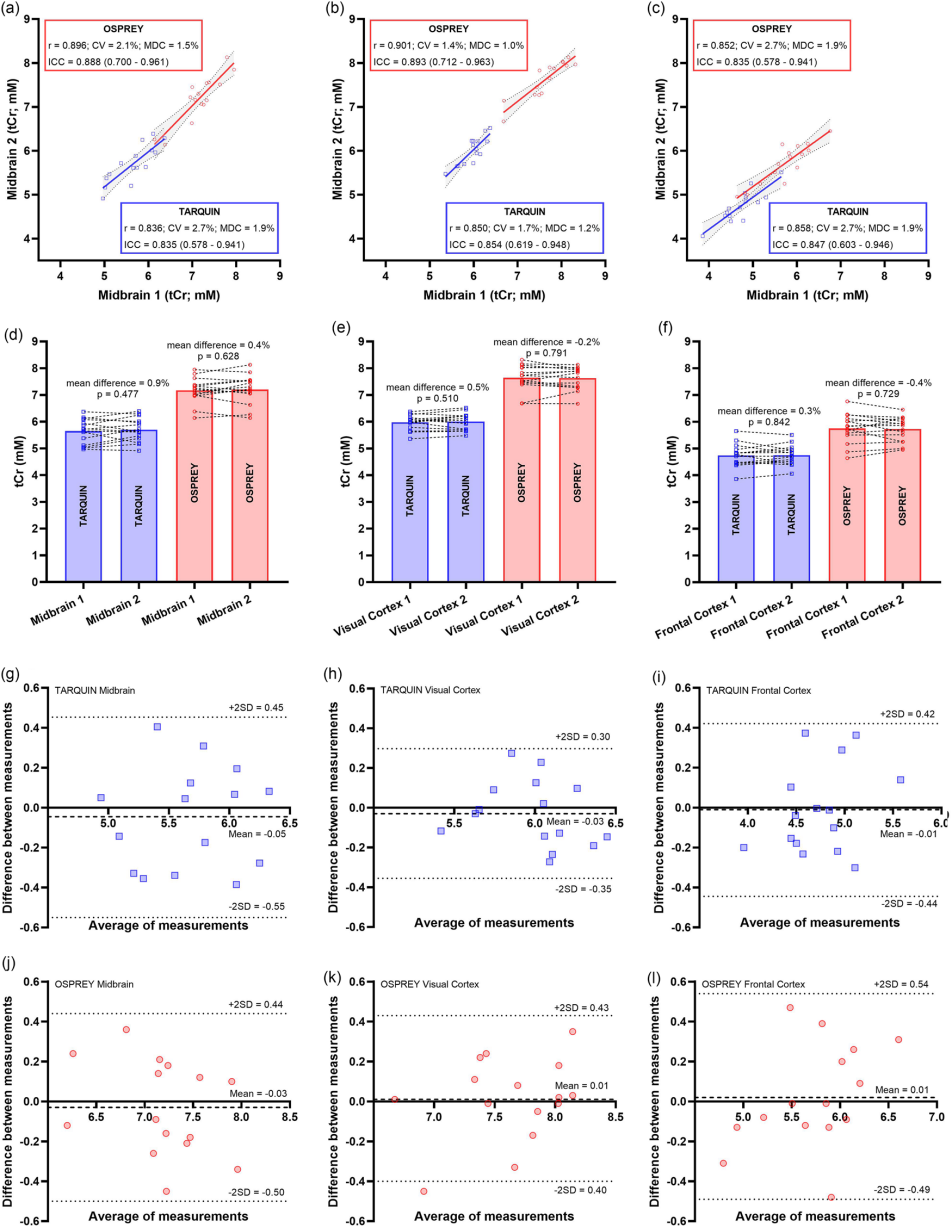
Inter‐session reliability and repeatability of ^1^H magnetic resonance spectroscopy for repeated total creatine (tCr) quantification in the midbrain (a, d, g, j), visual cortex (b, e, h, k) and frontal cortex (c, f, i, l) using TARQUIN (blue) or OSPREY (red). Pearson's correlation coefficients (*r*), coefficients of variation (CV), intra‐class correlation coefficients (ICC) and minimum detectable change (MDC) are shown (a–c), with the mean absolute differences (d–f) and Bland–Altman plots (g–l).

Strong to very strong Pearson's correlation coefficients were shown between repeated inter‐session tCr measurements for each of the brain regions, using either TARQUIN or OSPREY (for TARQUIN: MB, *r* = 0.836; VC, *r* = 0.850; and FC, *r* = 0.858; and for OSPREY: MB, *r* = 0.896; VC, *r* = 0.901; and FC, *r* = 0.852; all *P* < 0.001; Figure 6). No significant differences in mean absolute tCr concentrations were shown between repeated inter‐session measurements in any region, regardless of analysis package (for TARQUIN: MB measurement 1 vs. MB measurement 2 = 0.9%, *P* = 0.447; VC measurement 1 vs. VC measurement 2 = 0.5%, *P* = 0.510; and FC measurement 1 vs. FC measurement 2 = 0.3%, *P* = 0.842; and for OSPREY: MB measurement 1 vs. MB measurement 2 = 0.4%, *P* = 0.628; VC measurement 1 vs. VC measurement 2 = −0.2%, *P* = 0.791; and FC measurement 1 vs. FC measurement 2 = −0.4%, *P* = 0.729). For TARQUIN, the corresponding CVs were 2.7%, 1.7% and 2.7%, and the ICCs were 0.835 (95% CI = 0.578–0.941, *P* < 0.001), 0.854 (95% CI = 0.619–0.948, *P* < 0.001) and 0.847 (95% CI = 0.603–0.946, *P* < 0.001) for the VC, MB and FC, respectively. For OSPREY, the corresponding CVs were 2.1%, 1.4% and 2.7%, and the ICCs were 0.888 (95% CI = 0.700–0.961, *P* < 0.001), 0.893 (95% CI = 0.712–0.963, *P* < 0.001) and 0.835 (95% CI = 0.578–0.941, *P* < 0.001) for the VC, MB and FC respectively. Strong agreement between repeated inter‐session measurements was also indicated by the absence of trends in measurement error on the Bland–Altman plots (Figure 6). Minimum detectable changes were 1.9%, 1.2% and 1.9% (TARQUIN) and 1.5%, 1.0% and 1.9% (OSPREY) for the MB, VC and FC, respectively.

### 3.4 Differences in tCr concentrations according to brain region and analysis package

**FIGURE 7:**
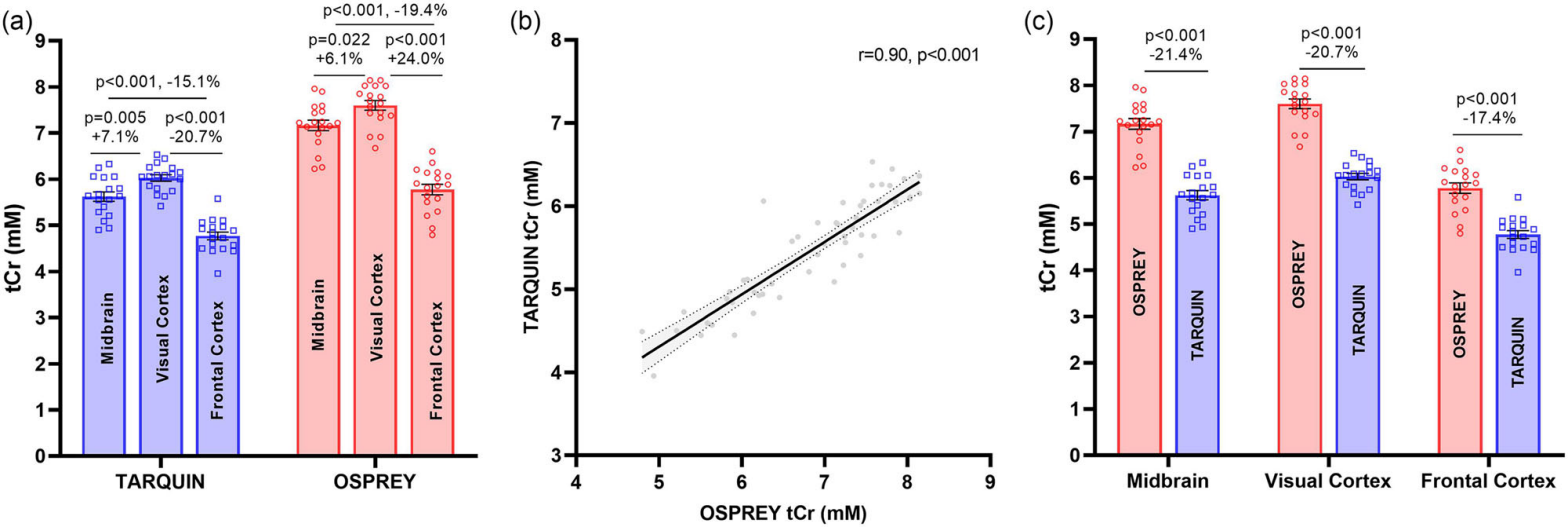
Differences in total creatine (tCr) concentrations according to brain region and analysis package (i.e., TARQUIN vs. OSPREY). (a) Inter‐regional differences in tCr concentration according to analysis package. (b) Correlation between tCr concentrations derived from TARQUIN versus OSPREY. (c) Region‐specific differences in tCr concentration according to analysis package. Mean values ± SEM are shown.

Across the full sample (*n* = 18), significant differences in tCr concentrations were shown between brain regions (using the average of the repeated measurements), regardless of the analysis package used (Figure 7). The greatest tCr concentrations were shown in the VC (TARQUIN, 6.02 ± 0.07 mM; OSPREY, 7.60 ± 0.10 mM), followed by the MB (TARQUIN, 5.62 ± 0.10 mM; OSPREY, 7.17 ± 0.11 mM), followed by the FC (TARQUIN, 4.77 ± 0.08 mM; OSPREY, 5.78 ± 0.11 mM). The specific differences using TARQUIN were 20.7% (1.25 mM) between the VC and FC, 15.1% (0.85 mM) between the MB and FC, and 7.1% (0.40 mM) between the VC and MB, and using OSPREY they were 24.0% (1.83 mM) between the VC and FC, 19.4% (1.39 mM) between the MB and FC, and 6.1% (0.43 mM) between the VC and MB. Although tCr concentrations determined using TARQUIN and OSPREY were very strongly correlated (*r* = 0.90, *P* < 0.001), the concentrations were consistently and substantially higher (between 17.4% and 21.4%) when determined using OSPREY, rather than TARQUIN (Figure 7).

## 4 DISCUSSION

The use of ^1^H MRS as a non‐invasive method of quantifying tCr levels in the brain will become increasingly prevalent in years to come, as interest in the role of Cr in supporting cognitive function increases and the efficiency and accessibility of MR improves. Importantly, however, the clinical value of ^1^H MRS is reliant upon the level of inherent measurement error, and large margins of error, which often encompass reported supplementation effects, permeate the existing literature. In light of this, we examined the reliability and repeatability of ^1^H MRS for repeated tCr quantification in the human brain using two state‐of‐the‐art analysis packages (i.e., TARQUIN and OSPREY). Encouragingly, our findings demonstrate that tCr concentrations can be determined accurately and repeatably, within an error of <2%, in multiple human brain regions when ^1^H MRS is acquired with a protocol that appropriately manages inherent sources of error and is processed using an automated analysis tool with adjustments to enhance consistency. Moreover, we provide new MDC data for tCr quantification by ^1^H MRS using this approach that might help to guide the interpretation of experimental studies.

Importantly, the present study was designed to allow for the specific determination of the inherent measurement error in repeated intra‐ and inter‐session PRESS‐based ^1^H MRS. Existing reliability and repeatability studies involved a considerable delay between repeated measurements (≤10 days) (Stephenson et al., 2011; Terpstra et al., 2016; Wang et al., 2024; Wijtenburg et al., 2013), which precludes the ability to distinguish the inherent margin of error in repeated ^1^H MRS from biological variations in tCr concentrations that might occur on a habitual basis. This disparity in study design might help to explain the poorer reliability and repeatability reported in these studies [e.g., CV of ≤7.5%, mean absolute differences of ≤10.2% according to the region (Wijtenburg et al., 2013), CV of 10% (Stephenson et al., 2011)] compared with the present study. Encouragingly, the reliability and repeatability of inter‐session ^1^H MRS achieved in our study is amongst the best reported to date at any magnetic field. Only two studies have reported somewhat comparable data, one conducted at 3 T [CV = 1.7%–4.4%, ICC = 0.64–0.82, mean absolute inter‐scan differences = 2.6%–6.3% (Wijtenburg et al., 2014)] and one conducted at 7 T [CV = 2.5%–3.8% depending on sequence (Okada et al., 2021)], although these studies focused solely upon the cingulate cortex and did not establish the MDC in tCr. As such, our study progresses current literature by incorporating a more appropriate protocol design (i.e., no delay between repeated measurements), several relevant brain regions, a cross‐comparison between two analysis packages, a range of reliability and reproducibility measures and an assessment of MDC.

Effective management of the sources of error in repeated ^1^H MRS is crucial for supporting the clinical value of the data. In future, therefore, researchers using ^1^H MRS to determine changes/differences in tCr concentrations should consider the following standardization measures: (1) ensure that voxel localization is performed by the same trained operator, using a high‐resolution structural scan to locate anatomical reference landmarks and using the previous assessment image to ensure accuracy of voxel relocalization for repeated measurements; (2) participants should be positioned comfortably, using additional cushioning around the head to promote stillness throughout the scan duration; (3) the same data acquisition parameters, analysis package (e.g., TARQUIN or OSPREY) and analysis package settings (e.g., lipid filtering, eddy‐current correction) should be used for repeated measurements; and (4) factors such as time of day, engagement in physical activity/exercise and sleep quality should be controlled for in experimental studies, until we have a better understanding of the effect of these variables on tCr concentrations in the brain. Analogous to the need for appropriate management of potential error sources is the need for more consistent and comprehensive reporting of methodological design for repeated ^1^H MRS studies. Such reporting should follow the recent consensus on reporting standards in MRS (Lin et al., 2021) and include detail of the efforts made to manage the sources of error mentioned above.

Before using ^1^H MRS to assess the response of tCr concentrations in the brain to various stimuli of interest, the inherent inter‐session margin of error and MDC need to be determined. This should be established in a centre‐specific manner incorporating an appropriate protocol, such as described in the present study. Notably, the limited availability of high‐quality ^1^H MRS reliability and repeatability data for tCr has been somewhat driven by the historical assumption that tCr concentrations are inherently stable in the brain (Li et al., 2003), and as a result, tCr has typically been reported as a denominator, rather than a numerator. Emerging data suggest, however, that brain tCr levels might be fluid, particularly in response to metabolically demanding periods (Candow et al., 2023; Forbes et al., 2022) and, as such, should be included as a metabolite of interest. Correspondingly, there is also a strong need to promote more consistent reporting of absolute tCr concentrations in ^1^H MRS studies, rather than arbitrary/institutional units or ratios that are frequently reported in existing literature. Doing so will allow for more comprehensive comparisons between studies and, ultimately, allow for a better understanding of the role of tCr in the human brain.

Interestingly, although we showed excellent reliability and repeatability statistics for all brain regions, significant inter‐regional differences were shown in spectral quality metrics. We showed that the MB had the poorest SNR, CRLB and FWHM, in comparison to the FC and VC. This aligns with previous reports of poorer quality spectra acquired from midbrain regions, such as the thalamus and hippocampus, in comparison to more distal regions, such as the dorsolateral prefrontal cortex and anterior cingulate cortex (Lind et al., 2020). The increased signal disturbance shown during ^1^H MRS of the midbrain might be somewhat explained by region‐specific differences in displacement and volumetric strain that occur during the cardiac and respiratory cycles, whereby areas of the midbrain are particularly susceptible to volumetric strain (Sloots et al., 2020) and are displaced to a greater degree than distal areas, such as the VC or FC (Almudayni et al., 2023). In the present study, the midbrain was the furthest examined region from the head coil, which might also explain the lower SNR compared with the other regions. Nevertheless, the average quality metrics from all brain regions were of suitable quality for absolute metabolite quantification (Wilson et al., 2019), which might help to contextualize the lack of inter‐regional differences in reliability and repeatability data despite differences in quality metrics.

Interestingly, no significant correlations were shown between changes in spectral quality metrics, voxel displacement or fractions, and changes in tCr concentration. It is noteworthy, however, that the small degree of inter‐session change in voxel fractions and displacement is a likely factor underpinning the lack of correlation shown in the present manuscript, and it is possible that larger inter‐session differences in these parameters would correlate with changes in tCr concentration. Moreover, although no significant associations were shown between change in spectral quality (i.e., CRLB, water FWHM and SNR) and variability in tCr concentration in the present study, it is possible that the cumulative effect of the changes in these parameters might have somewhat contributed to the within‐subject variability observed in tCr concentration. In future, therefore, more consistent and comprehensive reporting of spectral quality metrics, voxel relocalization procedures and tissue fractions will be central to improving our understanding of the impact that changes across these parameters have upon the quantification of tCr in the human brain.

It is also noteworthy that although we observed small intra‐subject variation in repeated tCr measurements [mean absolute differences ≤1.2% (TARQUIN) and ≤1.1% (OSPREY)], significant inter‐subject variation was shown for each brain region [up to 29.1%, 20.6% and 40.9% (TARQUIN) and up to 23.9%, 19.3% and 31.3% (OSPREY) in the MB, VC and FC, respectively], even within the present cohort of healthy young adults. This is an important consideration for future study design, because although small within‐subject effects might be detectable by ^1^H MRS (e.g., response to Cr supplementation), differences might need to be considerably larger to discern cohort‐specific differences in tCr concentrations confidently (e.g., older adults vs. younger adults). It is noteworthy, however, that the inter‐subject differences reported in the present study should be interpreted with consideration that part of the variation in tCr concentration might be attributable to factors such as time of day and sleep quality, although the effect of these variables on brain tCr concentrations remains to be determined. For example, given that some people took part in the morning and others in the late afternoon, it is plausible that the absolute differences in creatine shown between people could appear larger in the present manuscript than if we were to have scanned everyone at the same time of day. Notably, the inter‐subject variation in tCr concentration should be interpreted separately from the reliability and repeatability data, where the short intervals between measurements negated the need to control for variables such as time of day or sleep quality. There is an urgent need, therefore, to establish normative ranges of brain tCr concentrations in healthy adults across adulthood and to improve our understanding of the effect of time of day, sleep quality and prior activity. These data will enhance our understanding of the typical inter‐subject variation in tCr that is present during normal ageing and, ultimately, guide the interpretation of tCr concentrations in diseased cohorts or in those suffering from acute periods of metabolic stress.

Although not a primary aim of this study, we showed significant differences in tCr concentrations across each of the brain regions, with the highest concentrations in the VC, followed by the MB, then by the FC. Such inter‐regional differences are consistent with other reports suggesting that tCr concentrations differ according to brain region in healthy young and older adults (Lind et al., 2020) and in diseased populations (Hupfeld et al., 2023; Ongür et al., 2009). Importantly, our study builds upon these data by reporting inter‐regional differences in absolute tCr concentrations rather than institutional units, allowing for our data to be easily interpretable with future research. As discussed, more consistent reporting of absolute values is needed to promote inter‐study comparisons that are ultimately needed to progress in this field. Moreover, although accumulating data indicate that region‐specific differences in tCr concentrations exist, more research is needed to determine whether these differences are consistent at different stages of normal ageing and disease progression and whether they are associated with clinical outcomes relevant in these scenarios. This might help to illuminate, for example, mechanisms underpinning ageing and disease‐related phenotypes, and ultimately, identify potential therapeutic avenues.

It is particularly noteworthy that emerging data, which are now complemented by our findings, indicate that absolute metabolite quantities differ according to the analysis software used, even when identical ^1^H MRS data are used (Bhogal et al., 2017; Zöllner et al., 2021). Specifically, we show that although both analysis packages produce comparably precise data, the tCr concentrations quantified using TARQUIN were significantly lower (by between 17.4% and 21.4%) than those calculated using OSPREY, even within the present cohort of healthy young adults with high‐quality ^1^H MRS data. This inter‐software variance might be driven by differences in the modelling domain and fitting algorithm between OSPREY and TARQUIN. For example, there are inherent differences in the method of spectral processing between time‐domain modelling, upon which TARQUIN is based, and frequency‐domain modelling, upon which OSPREY is based. Time‐domain modelling analyses the ^1^H MRS data within the measurement domain, whereas frequency‐domain modelling integrates a frequency within which to analyse the data (0.5–4.0 ppm within OSPREY), which might help to explain the variance in tCr concentrations. Differences in baseline handling between TARQUIN and OSPREY (i.e., TARQUIN: first 10 ms of the free induction decay [FID] omitted and Gaussian window function of 100 data points applied vs. OSPREY: average of the 100 data points at the edges of the frequency‐domain spectrum is used, and default baseline knot spacing ranges from 0.15 ppm for preliminary modelling to 0.40 ppm for the full modelling procedure) might also contribute to the observed variance in tCr concentrations.

It is clear, therefore, that although a high level of precision in repeated tCr quantification in the human brain is possible, a level of uncertainty remains surrounding the accuracy of these data, given the substantial effect that choice of analysis package can have on tCr concentrations derived from identical MRS data. The future inter‐study comparison might, therefore, be limited by the analysis package used, hence improving our understanding of the consistency of tCr data across packages will be pivotal if a concerted effort is to be possible in this field. Until then, it is crucial that the same analysis package is used consistently within laboratories seeking to determine, for example, potential changes in brain tCr concentrations in response to stimuli, such as supplementation or sleep deprivation, or differences in brain tCr concentrations between metabolically demanding phenotypes, such as those induced by disease or ageing.

Several limitations to our work should be acknowledged. Firstly, only healthy young adults were included, and as such, our findings might not be generalizable to other cohorts who might have more diverse ranges in tCr concentrations. Accordingly, future studies incorporating aged or diseased participants are needed to determine whether similar accuracy can be achieved in other populations. Secondly, including three repeated measurements, rather than two, might have provided a more robust assessment of reliability and repeatability, although this would have increased scan duration and possibly reduced compliance. Thirdly, although the purpose of this study was to examine ^1^H MRS, the Cr concentration attained from this technique represents the sum of free Cr and PCr, hence future reliability studies using phosphorus MRS (^31^P MRS) are warranted to determine the reliability of repeated PCr quantification. Finally, on a similar but more general note, studies incorporating both ^1^H MRS and ^31^P MRS are ultimately needed to provide a more thorough overview of Cr dynamics in the human brain.

## 5 CONCLUSION

In conclusion, we demonstrate that when an appropriate protocol is adopted, ^1^H MRS at 3 T can determine absolute tCr concentrations in multiple regions of the human brain with an excellent level of reliability and repeatability. Changes in tCr concentration need to be ≥2% to be discernible from ^1^H MRS measurement error. Future studies should seek to determine whether these findings can be replicated in other centres and in other populations of interest. In this regard, more consideration needs to be given to the inherent sources of error in ^1^H MRS, including the choice of analysis package, before we can accurately interpret data from experimental studies in this exciting field.

## AUTHOR CONTRIBUTIONS

Jedd Pratt designed the work, acquired, analysed and interpreted the data and drafted the manuscript. James McStravick acquired the data and critically revised the manuscript. Aneurin J. Kennerley designed the work, acquired, analysed and interpreted the data and critically revised the manuscript. Craig Sale conceived the idea for the work, interpreted the data and critically revised the manuscript. All authors approved the final version of the manuscript and agree to be accountable for all aspects of the work in ensuring that questions related to the accuracy or integrity of any part of the work are appropriately investigated and resolved. All persons designated as authors qualify for authorship, and all those who qualify for authorship are listed.

## CONFLICT OF INTEREST

All authors declare no competing interests.

## FUNDING INFORMATION

None.

## Supporting information



Supplementary Material

## Data Availability

Data may be made available upon reasonable request to the corresponding author (C.S.).
